# The PI3K/Akt Pathway in Herpesvirus Biology: A Double-Edged Sword in Host–Virus Interactions

**DOI:** 10.3390/microorganisms14020337

**Published:** 2026-02-02

**Authors:** Divya Kapoor, Pankaj Sharma, Mannat Singh, Deepak Shukla

**Affiliations:** 1 Department of Microbiology, Immunology and Inflammation, University of Illinois at Chicago, Chicago, IL 60612, USA; dkapoo5@uic.edu; 2 Department of Ophthalmology and Visual Sciences, University of Illinois at Chicago, Chicago, IL 60612, USA; sharmap@uic.edu (P.S.); msing7@uic.edu (M.S.)

**Keywords:** human herpesviruses, PI3K/Akt pathway, therapeutic, inflammation, disease

## Abstract

Human herpesviruses (HHVs) are notorious, ubiquitous intracellular pathogens that establish lifelong infections in the host. They tightly manipulate host signaling pathways that play central roles in key cellular processes such as cell survival, metabolism, immune responses, and oncogenic transformation. Among the many pathways explored, the phosphatidylinositol-3-kinase (PI3K)/Akt signaling axis has emerged as a central and conserved target exploited by all eight HHVs. Herpesviruses can induce PI3K/Akt signaling at multiple stages of their life cycle, beginning at viral entry and extending through lytic replication, latency maintenance, immune evasion, and virus-associated tumorigenesis. Mechanistically, herpesviruses engage both host cell receptors and viral effector proteins to activate PI3K, drive Akt phosphorylation, and thereby orchestrate downstream signaling pathways that favor viral replication, survival, and immune evasion. Transient activation of this pathway supports viral replication, whereas sustained signaling promotes latent infection and oncogenesis, particularly in Epstein–Barr virus and Kaposi’s sarcoma-associated herpesvirus. This review provides a comparative analysis of PI3K/Akt pathway manipulation across all HHVs, highlighting shared strategies and virus-specific adaptations. We further discuss ongoing clinical trials and therapeutic opportunities targeting the PI3K/Akt axis, emphasizing its potential as a host-directed antiviral and anticancer strategy.

## 1. Introduction

HHVs represent an important class of medically important human pathogens that establish lifelong infections. They can cause wide spectrum of diseases extending from mucocutaneous lesions to severe ocular disease like Herpetic Stromal Keratitis (HSK), encephalitis, congenital infections, and virus-associated malignancies [1,2]. Majority of the global population is infected by one or multiple members of the Herpesviridae family, including herpes simplex virus (HSV) type 1 and 2, varicella–zoster virus (VZV), cytomegalovirus (CMV), Epstein–Barr virus (EBV), and Kaposi’s sarcoma-associated herpesvirus (KSHV) [3]. Herpesviruses are unique in their ability to transition between lytic replication and latency and therefore require tight control over host cellular pathways to support viral persistence, reactivation, and immune evasion. Despite the availability of antiviral therapies that primarily target viral replication, herpesvirus-associated diseases continue to impose a substantial clinical burden. This underscores the need for a deeper understanding of virus–host interactions that can be strategically targeted to enable the development of more effective and efficient therapeutic interventions [4]. Herpesviruses are obligate pathogens, so they rely entirely on host cellular machinery to support viral gene expression, genome replication, and virion assembly. Herpesviruses capture the host machinery completely to create a cellular environment favorable for their own survival beyond exploiting basic biosynthetic processes such as protein translation and nucleotide synthesis [5,6]. There are multiple intracellular signaling pathways including metabolism, cell cycle progression, apoptosis, and innate immune responses that directly shape herpesvirus replication, persistence, and latency. Among these pathways, protein phosphorylation cascades have emerged as critical nodes that herpesviruses co-opt to coordinate viral gene expression, inhibit premature cell death, and modulate antiviral defenses [7].

Herpesviruses impose selective pressure on host cellular machinery to precisely activate host kinases that promote viral replication while escaping or suppressing pathways that trigger antiviral responses [8]. For this purpose, herpesviruses deploy specific viral proteins that selectively activate host kinases and phosphatases to fine-tune signaling outputs [9]. This specificity in terms of selectively activating certain host kinases is important as many kinases can also restrict viral replication through induction of apoptosis, interferon signaling, or inflammatory responses. Eventually, herpesviruses are vastly known to evolved themselves with the host in a sophisticated manner to bias host signaling toward proviral outcomes while dampening host defense pathways. In this aspect, the phosphatidylinositol-3-kinase (PI3K)/Akt signaling axis has consistently surfaced as a central and recurrent target during herpesvirus infection. Moreover, this pathway is a master regulator of all the key processes for herpesvirus replication and persistence including cell survival, metabolism, protein synthesis, and stress responses. Activation of PI3K/Akt signaling during herpesvirus infection has been reported to enhance viral entry, promote viral protein translation, suppress apoptosis, and support latency establishment and reactivation. At the same time, PI3K/Akt signaling intersects with innate immune pathways, positioning it as a critical interface between viral replication and host defense [10,11].

Other than herpesviruses, diverse range of viruses exploit the PI3K/Akt signaling pathway, underscoring its central role as a conserved host signaling hub during their life cycle. Many RNA and DNA viruses exploit this pathway at different steps during their phase of infection ranging from early stages to promote viral entry, suppress apoptosis, upto their efficient replication and spread [12]. For example, influenza A virus [13] and respiratory syncytial virus transiently activate PI3K/Akt to delay host cell death and prolong viral production, while hepatitis C virus [14] and dengue virus [15] use sustained Akt signaling to remodel cellular metabolism and lipid synthesis required for replication complex formation. Several oncogenic viruses also activate this pathway including human papillomavirus and hepatitis B virus, chronically engage PI3K/Akt signaling to enhance cell survival, proliferation, and immune evasion, contributing to virus-associated malignancies. In contrast to herpesviruses, which uniquely integrate PI3K/Akt signaling into latency establishment and long-term persistence, many acute viruses primarily rely on this pathway to optimize short-term replication and spread. Together, these observations highlight PI3K/Akt as a broadly targeted host pathway across viral infections, emphasizing its relevance beyond herpesvirus biology and reinforcing its potential as a therapeutic target.

Notably, PI3K/Akt signaling functions as a double-edged sword during different herpesvirus infection. In many herpesviruses’ infection, PI3K/Akt activation supports their replicative and latent programs but, in some cases, it can also contribute to antiviral responses which is based on the cellular context, timing, and magnitude of activation [16,17]. This dual role highlights the complexity of PI3K/Akt signaling in herpesvirus biology and emphasizes its importance as both a proviral and host-protective pathway. This review focuses on the mechanisms by which herpesviruses manipulate the PI3K/Akt signaling cascade throughout different stages of infection. We discuss how diverse herpesviruses converge on this pathway using distinct viral strategies, the consequences of PI3K/Akt modulation for viral replication, latency, and immune evasion, and the implications of this signaling axis as a potential target for host-directed antiviral therapies.

## 2. Overview of the PI3K/AKT Pathway

**Figure 1:**
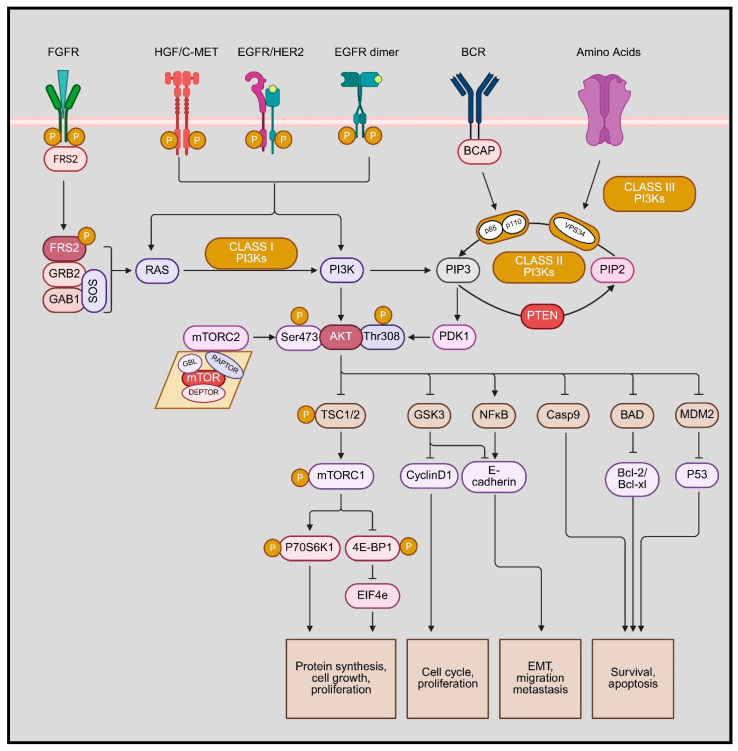
Overview of PI3K/Akt signaling pathway (created in BioRender. Kapoor, D. (2026) https://BioRender.com/r0srkqg).

**Figure 2:**
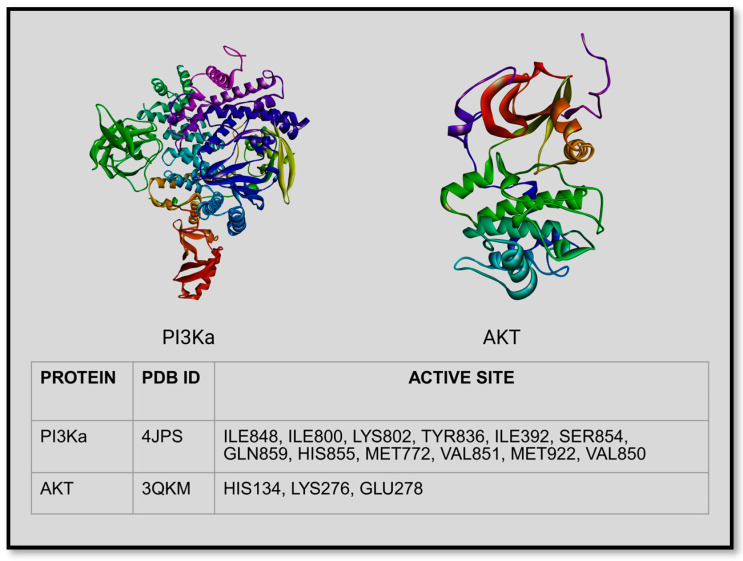
Structure of PI3K and AKT indicating their active sites (created in BioRender. Kapoor, D. (2026) https://BioRender.com/s8fuvtt).

The phosphoinositide 3-kinase (PI3K)/Akt signal transduction axis is a key intracellular signaling pathway that regulates cell survival, growth, metabolism, proliferation, and stress responses [18]. It is a classical phosphorylation cascade that utilizes tyrosine, lipid, and serine–threonine phosphorylation to transduce extracellular cues to internal responses. Activation of the pathway typically begins at the cell surface when ligands bind receptor tyrosine kinases (RTKs), G-protein coupled receptors, or integrins. This leads to recruitment and activation of PI3K, which phosphorylates the membrane lipid phosphatidylinositol-4,5-bisphosphate (PIP2) to generate phosphatidylinositol-3,4,5-trisphosphate (PIP3). PIP3 functions as a docking platform at the plasma membrane, allowing the recruitment of Akt (protein kinase B) and its upstream activator PDK1 [19]. Once localized to the membrane, AKT becomes fully activated through phosphorylation at two key residues by PDK1 and mTORC2. Activated AKT then phosphorylates a broad range of downstream targets that collectively promote cell survival by inhibiting apoptosis, enhance protein synthesis and growth, stimulate glucose uptake and metabolism, and regulate cell cycle progression. One of the most important downstream branches involves activation of mTORC1, linking PI3K/AKT signaling to anabolic metabolism and autophagy regulation [20]. The pathway is tightly controlled by negative regulators, most notably PTEN, a lipid phosphatase that dephosphorylates PIP3 back to PIP2, thereby terminating signaling (Figure 1). Loss or dysregulation of this control leads to sustained PI3K/AKT activation, which is commonly associated with cancer, chronic inflammation, metabolic disorders, and viral infections. Because of its central role in coordinating survival and inflammatory signaling, the PI3K/AKT pathway is a major therapeutic target and a critical node in host–pathogen interactions [21]. Figure 2 depicts the structural organization of PI3K and AKT, highlighting PI3K’s major catalytic and regulatory active sites to facilitate understanding of pharmacological targeting [22].

Other than viruses a wide range of bacteria also engage this pathway during bacterial pathogenesis, the PI3K/Akt pathway is widely co-opted to facilitate its spread and immune evasion, but its functional deployment differs in important ways from viral infections [23,24,25]. Several intracellular bacterial pathogens, including *Salmonella enterica*, *Listeria monocytogenes*, *Shigella flexneri*, and *Mycobacterium tuberculosis*, actively manipulate host PI3K/Akt signaling to facilitate cellular entry. This activation occurs either through the delivery of bacterial effector proteins via specialized secretion systems or through engagement of host surface receptors, leading to localized PI3K activation at the plasma membrane. Subsequent Akt signaling orchestrates extensive remodeling of the actin cytoskeleton by regulating small Rho GTPases and actin-associated proteins, resulting in membrane ruffling, cytoskeletal rearrangement, and engulfment of the pathogen. Through this mechanism, bacteria are able to promote both phagocytic uptake by professional immune cells and induced internalization into non-phagocytic epithelial cells, thereby establishing an intracellular niche essential for their survival and persistence [26,27,28,29]. Once internalized, sustained Akt activation suppresses apoptosis and modulates autophagy, allowing bacteria to persist within membrane-bound compartments or cytosolic niches. In macrophages, PI3K/Akt signaling further dampens antimicrobial responses by limiting inflammasome activation, nitric oxide production, and pro-inflammatory cytokine release. In contrast, herpesviruses exploit PI3K/Akt not only during entry but across multiple stages of their life cycle, including genome replication, inhibition of premature cell death, immune evasion, and maintenance of latent infection [21]. Viral activation of PI3K/Akt is often more prolonged and tightly integrated with transcriptional and epigenetic reprogramming of the host cell, particularly in long-lived reservoirs such as neurons and lymphocytes. Thus, while both bacterial and viral pathogens converge on PI3K/Akt as a central regulator of cell survival, metabolism, and innate immunity, bacterial pathogens primarily use this pathway to support acute intracellular persistence and niche stabilization, whereas herpesviruses harness PI3K/Akt to enable long-term persistence, latency, and reactivation, underscoring its role as a shared but differentially exploited host signaling hub.

### 2.1. Downstream Effectors and Cellular Functions

Akt upon activation further phosphorylates a broad array of substrates that influence key cellular processes. A major target is the tuberous sclerosis complex (TSC1-TSC2). TSC2 (tuberin) functions together with TSC1 (hamartin) as a tumor suppressor complex that negatively regulates mTOR complex 1 (mTORC1). Under basal conditions, the TSC1-TSC2 complex acts as a GTPase-activating protein (GAP) for the small GTPase Rheb, converting active Rheb-GTP into inactive Rheb-GDP. Because Rheb-GTP is a direct activator of mTORC1, this GAP activity keeps mTORC1 signaling suppressed. When AKT is activated, it phosphorylates TSC2 at multiple regulatory serine and threonine residues. This phosphorylation inhibits the tumor suppressor function of the TSC1-TSC2 complex by disrupting its GAP activity toward Rheb and promoting TSC2 sequestration away from its site of action. As a result, Rheb remains in its GTP-bound active state, which directly stimulates mTORC1 kinase activity. Thus, AKT-mediated phosphorylation of TSC2 relieves the inhibitory brake imposed by the TSC1-TSC2 complex on mTORC1, leading to enhanced mTORC1 signaling that promotes protein synthesis, cell growth, and metabolic reprogramming [30]. This activation of mTORC1 further activates its downstream effectors, such as ribosomal S6 kinase (S6K) and 4E-BP1, which drive protein synthesis, nutrient uptake, and cell growth/proliferation [30].

Other than indirectly modulating multiple signaling cascades, AKT also directly modulates metabolism; for example, it inactivates glycogen synthase kinase-3 (GSK-3) and promotes glucose uptake and glycolysis (in part through regulators of GLUT4 trafficking), linking PI3K/AKT signaling to metabolic control [31]. Interestingly, PI3K/AKT signaling promotes cell survival and opposes apoptosis. AKT can phosphorylate and inactivate several pro-apoptotic proteins. For instance, AKT phosphorylates the Bcl-2 family member BAD, preventing BAD from binding Bcl-xL and thereby blocking BAD-induced cell death [32]. Furthermore, AKT also phosphorylates and inhibits components of the intrinsic apoptosis pathway such as caspase-9, as well as key transcription factors of the Forkhead box O (FOXO) family. Phosphorylation of FOXO proteins by AKT causes their sequestration in the cytoplasm (via 14-3-3 protein binding), thereby preventing FOXO from inducing pro-apoptotic genes in the nucleus [32]. Through these actions, activated AKT potently enhances cell survival and proliferation. Indeed, fully active AKT simultaneously influences angiogenesis, metabolism, growth, proliferation, and survival programs, illustrating its role as a pleiotropic regulator of cellular physiology.

### 2.2. Integration and Crosstalk in Signaling Networks

As mentioned earlier, the PI3K/AKT pathway functions as a central node in intracellular signaling by incorporating inputs from various upstream complex pathways and interfacing with other key pathways. PI3K/Akt pathway can be stimulated by various means other than RTKs, including integrin-mediated adhesion, G-protein coupled receptors, cytokine receptors, and antigen receptors, reflecting the pathway’s broad role in sensing the extracellular environment. In this signaling landscape, the Ras-MAPK pathway is a notable partner: the small GTPase Ras directly binds the p110α catalytic subunit of PI3K, providing an alternate route to PI3K activation in parallel to Ras’s stimulation of the Raf/MEK/ERK cascade [33]. This direct Ras-PI3K interaction plays an important role in normal growth factor signaling and also triggers full Ras-driven oncogenic transformation in vivo. These pathways are multidirectional and the crosstalk is evident, for example, some MAPK pathway components can impinge on PI3K/AKT signaling underscoring that these pathways do not operate in isolation. Though, PI3K/Akt still remains a central node of this highly complex network that coordinates with other signaling modules to fine-tune numerous outcomes like cell cycle progression and differentiation in response to growth cues or stress [34].

### 2.3. Regulation and Implications of Dysregulation

The PI3K/Akt pathway is subject to strict negative regulations owing to its robust pro-growth and pro-survival outputs. Several tumor suppressors act on this pathway: PTEN (phosphatase and tensin homolog) is a lipid phosphatase that directly opposes PI3K by dephosphorylating PIP3 back to PIP2, thus attenuating Akt recruitment and activation [35]. Additionally, serine/threonine phosphatases like PP2A and the PHLPP1/2 phosphatases deactivate Akt by removing the activating phosphorylation (Thr308 and Ser473, respectively). Through these mechanisms, cells ensure that Akt signaling is transient and appropriately checked once an upstream signal dissipates.

Loss of proper PI3K/Akt regulation is a hallmark of many diseases, especially cancer. Oncogenic mutations frequently hyperactivate this pathway, for example, activating mutations in the PIK3CA gene (encoding the p110α subunit of PI3K) occur in a significant fraction of human tumors, and AKT itself can be activated by amplification or point mutation in some cancers. Conversely, PTEN is one of the most commonly lost tumor suppressors in sporadic cancers; PTEN inactivation results in unchecked accumulation of PIP3 and constitutive AKT signaling. The net effect of such dysregulation is enhanced cell proliferation and survival, contributing to oncogenesis. Beyond cancer, viruses often hijack the PI3K/Akt pathway to benefit their replication. For instance, hepatitis C virus (HCV) has been shown to rapidly and transiently activate Akt within minutes of infecting a cell, which facilitates viral entry and protects the infected cell from apoptosis inhibition of PI3K/Akt significantly impairs HCV infection [36]. Many other viruses (e.g., vaccinia, hepatitis B, various RNA viruses, and herpesviruses) similarly activate PI3K/Akt signaling in host cells to promote cell survival or metabolic conditions favorable for viral replication. In summary, tight control of the PI3K/Akt pathway is critical for normal physiology, and its dysregulation whether by genetic mutations in cancers or subversion by viral proteins can lead to pathological cell survival, uncontrolled growth, and disease progression [35].

## 3. General Biology of Herpesviruses

Herpesviruses are large, enveloped viruses with linear double-stranded DNA genomes that have evolved an extraordinary capacity for lifelong persistence in their hosts. More than 100 herpesviruses have been identified across vertebrate species, of which eight are known to infect humans [37]. A defining feature shared by all human herpesviruses (HHVs) is their ability to establish latency following primary infection, allowing the viral genome to persist in specific host cell types for the lifetime of the individual. Rather than maintaining continuous replication, herpesviruses alternate between productive lytic infection and transcriptionally restricted latency, a strategy that enables immune evasion while preserving the potential for reactivation under favorable conditions.

Epidemiologically, herpesviruses are among the most successful human pathogens. A substantial proportion of the global population harbors at least one HHV, often asymptomatically. HSV-1 alone infects billions of individuals worldwide. Current estimates indicate that approximately 3.7 to 3.8 billion people are infected with HSV-1, while 491 to 520 million carry HSV-2. EBV infects more than 90 percent of adults worldwide, and CMV seroprevalence ranges from 40 to nearly 100 percent depending on geographic and socioeconomic factors. VZV seropositivity exceeds 90 percent in many populations, and HHV-6 and HHV-7 infect the majority of children and persist in adulthood [38]. The widespread distribution of these viruses underscores their efficient transmission and long-term coexistence with the human host.

### 3.1. Virion Structure and Genome Organization

Despite their biological diversity, all herpesviruses share a conserved virion architecture composed of four distinct structural layers. At the center lies a large linear double-stranded DNA genome, ranging approximately from 120 to 250 kilobase pairs depending on the virus [39]. This genome is enclosed within an icosahedral capsid composed of 162 capsomers arranged with strict symmetry. Surrounding the capsid is the tegument, an amorphous protein-rich layer that contains viral factors essential for early stages of infection, immune modulation, and intracellular trafficking. The outermost layer is a host-derived lipid bilayer envelope embedded with multiple viral glycoproteins that mediate attachment, entry, and cell-to-cell spread.

Herpesvirus genomes exhibit complex arrangements of unique and repeated sequences that differ among subfamilies. These structural variations allow genome isomerization and contribute to genetic diversity during replication. In some herpesviruses, terminal and internal repeat regions divide the genome into distinct segments that can exist in multiple equimolar configurations. Such genome flexibility is thought to enhance adaptability and may influence latency, recombination, and reactivation dynamics [40].

### 3.2. Classification of Human Herpesviruses

Based on genomic structure, replication kinetics, host range, and latency reservoirs, the Herpesviridae family is divided into three major subfamilies: Alphaherpesvirinae, Betaherpesvirinae, and Gammaherpesvirinae [41,42,43,44].

Alphaherpesviruses, which include HSV-1, HSV-2, and varicella–zoster virus, are characterized by rapid replication cycles and pronounced cytopathic effects in infected epithelial cells. These viruses exhibit a broad host range and establish lifelong latency primarily in sensory neurons. During latency, viral genomes persist episomally with highly restricted transcription, allowing neuronal survival while maintaining the potential for reactivation. Reactivation leads to recurrent disease at peripheral sites and, in some cases, severe ocular or neurological complications [45,46].

Betaherpesviruses, including human cytomegalovirus and HHV-6 and HHV-7, replicate more slowly and display a narrower host cell range. Infection is often associated with enlarged, cytomegalic cells in vitro. These viruses establish latency in cells of the myeloid lineage, hematopoietic progenitors, and secretory tissues, enabling systemic persistence. Reactivation of betaherpesviruses is closely linked to immunosuppression, inflammation, or cellular differentiation and is a major cause of morbidity in transplant recipients and other immunocompromised populations [47].

Gammaherpesviruses, represented by Epstein–Barr virus and Kaposi’s sarcoma-associated herpesvirus, exhibit the most restricted host range and preferentially infect lymphoid cells. Latency is maintained through tightly regulated transcriptional programs involving viral proteins and noncoding RNAs that manipulate host chromatin, immune signaling, and cell survival pathways. Persistent gammaherpesvirus infection is strongly associated with lymphoproliferative disorders and virus-driven malignancies [48,49].

### 3.3. Replication Cycle and Temporal Gene Expression

Herpesvirus replication is a multistep process that is spatially and temporally regulated within the host cell. Following viral entry, the capsid is transported to the nucleus, where the viral genome is released and circularized. The host cell nucleus serves as the central hub for viral transcription, genome replication, and capsid assembly. Viral gene expression follows a conserved temporal cascade consisting of immediate-early, early, and late phases. Immediate-early genes encode regulatory proteins that reprogram host transcription and suppress antiviral defenses. Early genes support viral DNA synthesis, while late genes encode structural components required for virion assembly [50,51].

Assembly of the nucleocapsid occurs within the nucleus, after which the virus acquires its tegument and envelope through interactions with nuclear and cytoplasmic membranes, including the endoplasmic reticulum and Golgi apparatus [52]. Mature virions are transported to the plasma membrane within vesicles and released from the cell, a process that is frequently accompanied by host cell death. Notably, herpesvirus replication is relatively inefficient, producing a high proportion of noninfectious particles alongside infectious virions [53,54,55,56,57,58,59,60].

### 3.4. Latency and Reactivation

Latency is the hallmark of herpesvirus biology and represents an actively maintained state rather than viral dormancy. During latency, viral genomes persist within the nucleus either as episomes or, in rare cases, integrated forms, with minimal expression of lytic genes [61,62,63,64,65,66]. This transcriptional silencing limits immune recognition while preserving the viral genome. Each herpesvirus establishes latency in a characteristic cell type, including neurons, lymphocytes, monocytes, and progenitor cells, reflecting virus-specific tissue tropism [67,68].

Reactivation from latency can occur in response to diverse physiological and environmental stimuli, including cellular stress, inflammation, immune suppression, hormonal fluctuations, and ultraviolet exposure. Reactivation involves epigenetic remodeling of viral chromatin and reinitiation of immediate-early gene expression, allowing the virus to reenter the lytic cycle. While reactivation may be asymptomatic, it can also result in recurrent disease or life-threatening pathology, particularly in immunocompromised hosts [69,70]. The precise molecular mechanisms governing latency maintenance and reactivation remain incompletely understood, highlighting a critical area of ongoing herpesvirus research.

## 4. Host Immune Evasion Strategies

Herpesviruses persist within immunocompetent hosts by deploying multilayered immune evasion strategies that target both innate and adaptive immune responses. These mechanisms operate throughout the viral life cycle and are tailored to different stages of infection, including lytic replication, latency, and reactivation. Rather than relying on a single pathway, herpesviruses interfere with immune detection at multiple checkpoints, allowing them to establish stable, lifelong infections. At the level of innate immunity, herpesviruses disrupt early pathogen recognition and antiviral signaling. Viral proteins delivered in the tegument immediately upon entry can inhibit cytosolic and endosomal sensors responsible for detecting viral nucleic acids, thereby delaying the induction of type I interferon responses. Additional viral factors interfere with downstream signaling cascades, including transcription factors required for interferon-stimulated gene expression. This early suppression of innate immunity creates a permissive intracellular environment that supports viral gene expression and replication before robust host defenses are mobilized. Such interference is conserved across herpesvirus subfamilies, underscoring its importance for successful infection [71,72]. Herpesviruses also extensively target adaptive immune responses, particularly antigen presentation to cytotoxic T lymphocytes. Many herpesviruses encode proteins that disrupt the processing, trafficking, or surface expression of major histocompatibility complex (MHC) class I molecules. By retaining MHC molecules within intracellular compartments or targeting them for degradation, infected cells display fewer viral peptides at the cell surface, reducing recognition by CD8^+^ T cells.

To compensate for the resulting vulnerability to natural killer (NK) cell mediated killing, herpesviruses simultaneously modulate NK cell activating and inhibitory ligands, effectively balancing evasion of both arms of cellular immunity. These mechanisms are especially prominent during lytic infection, when viral antigen levels would otherwise trigger rapid immune clearance [73,74]. Latency represents the most effective and durable immune evasion strategy employed by herpesviruses. During latent infection, viral gene expression is highly restricted, minimizing antigen availability and rendering infected cells largely invisible to immune surveillance. Latency is maintained through epigenetic silencing of lytic gene promoters and continuous regulation of host transcriptional machinery. Importantly, latency is not immunologically inert. In gammaherpesviruses, latent proteins and noncoding RNAs actively reshape host immune signaling pathways, dampening antiviral responses while promoting infected cell survival and proliferation. These processes allow long-term persistence while preserving the ability to reactivate when host conditions permit [52,64,75]. In addition to intracellular immune modulation, herpesviruses alter the extracellular environment to facilitate persistence and spread. Viral infection can induce remodeling of the extracellular matrix, modulate cytokine and chemokine gradients, and influence immune cell recruitment to infected tissues. These changes can promote viral dissemination while simultaneously exacerbating inflammation and tissue damage during recurrent disease. Such indirect immune modulation highlights how herpesviruses shape not only infected cells but also the broader tissue microenvironment to their advantage [71,76]. Collectively, these immune evasion strategies enable herpesviruses to establish a finely tuned equilibrium with the host immune system. By suppressing immune detection during lytic infection, minimizing antigen expression during latency, and selectively reengaging immune pathways during reactivation, herpesviruses achieve long-term persistence while retaining pathogenic potential. These interactions between viral immune evasion mechanisms and host regulatory pathways provide critical context for understanding how cellular signaling networks are co-opted during herpesvirus infection.

## 5. Importance of PI3K/AKT in Viral Pathogenesis

**Figure 3:**
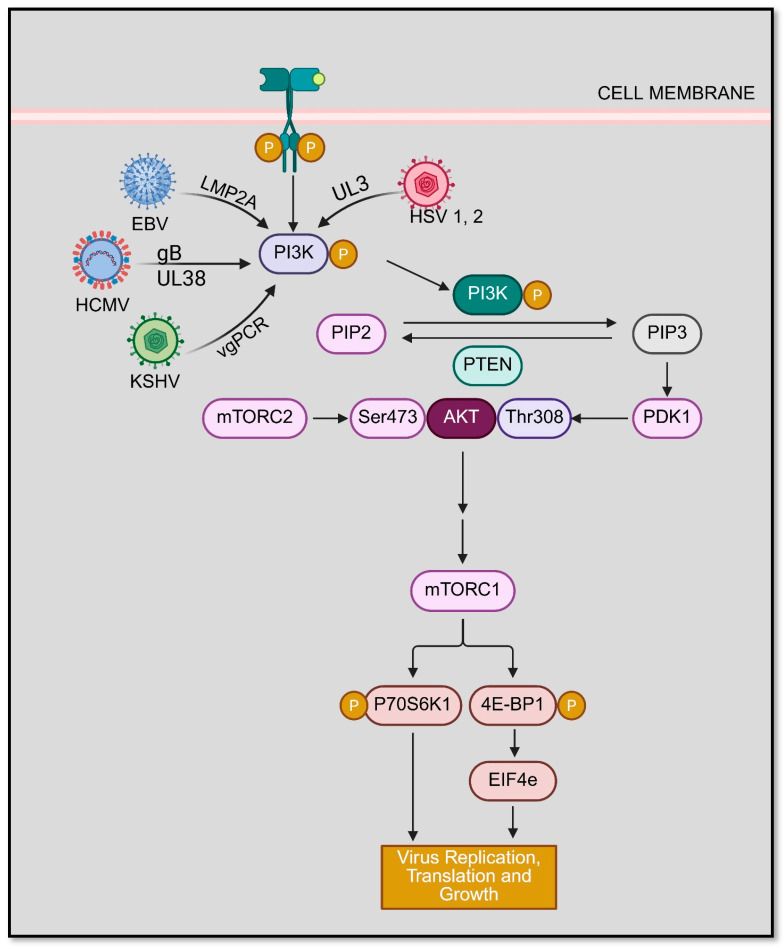
Activation of Akt signaling by Herpesviruses (created in BioRender. Kapoor, D. (2026) https://BioRender.com/r2k0r9b).

The PI3K/Akt signaling axis plays a central role in the regulation of cell survival, metabolism, and immune responses. All HHVs, from HSV-1/2 to HCMV, EBV, including others have evolved different strategies to manipulate and control PI3K/Akt signaling during infection (Figure 3) [77]. The successful activation and modulation of this pathway aids in viral replication and latency establishment while subverting antiviral [77]. The virus-induced manipulation of this pathway is a double-edged sword as it prolongs the cell survival (benefiting viral persistence) and dampens the immune responses; alternatively, the chronic PI3K/Akt activation potentially contributes to oncogenic transformation in infected cells [77]. In the upcoming subsections, we summarize how these HHVs hijack and manipulate the PI3K/Akt axis, emphasizing the dual roles in immune modulation and virus-driven oncogenesis.

### 5.1. PI3K/AKT Activation in Herpesvirus Infection and Immune Modulation

Alphaherpesviruses (HSV-1, HSV-2, VZV): The infection with HSV-1/2 leads to a rapid activation of this pathway to enhance viral entry, block cell death, and counter antiviral responses [78,79]. Consistent with the literature on corneal viral disease, modulation of focal adhesion kinase (FAK)/PI3K/Akt signaling has been observed in corneal infectious models, suggesting that this pathway contributes to host tissue responses and inflammatory signaling during ocular herpesvirus pathogenesis [80]. The viral factor VP11/12 (gene UL46), tegument protein, plays a crucial role in this process. It functions as a constitutively active receptor surrogate by recruiting Src family kinases and engaging in the p85 regulatory subunit of PI3K, thereby eliciting robust Akt activation during the early stages of infection [78]. In parallel, the viral serine/threonine kinase US3, a conserved protein among alphaherpesviruses, directly phosphorylates canonical pro-apoptotic Akt substrates, effectively serving as an Akt functional analog to promote survival of infected cells. Together, these strategies allow HSV to suppress apoptotic pathways and dampen innate antiviral signaling, creating a permissive intracellular milieu that supports viral gene expression and replication. VZV employs a closely related strategy through its ORF12 protein, which directly associates with the p85 regulatory subunit of PI3K to potently activate Akt signaling, thereby modulating host cell-cycle progression [81]. ORF12-driven Akt activation elevates cyclin expression and drives cells out of G1 arrest, facilitating productive VZV replication in both proliferating epidermal cells and post-mitotic neurons. Consistent with this central role, pharmacological inhibition of the PI3K/Akt pathway markedly suppresses VZV replication, underscoring the pathway’s critical importance in alphaherpesvirus infection.

Betaherpesviruses (HCMV, HHV-6, HHV-7): HCMV extensively exploits the PI3K/Akt pathway at multiple stages of its life cycle. During viral entry, HCMV envelops glycoproteins, including gB and the gH/gL complex, engage cellular receptors such as EGFR and integrins, triggering PI3K/Akt signaling that is required for efficient viral internalization [82]. Infected monocytes that are short-lived cells are co-opted by HCMV as “Trojan horses” for systemic dissemination. The virus activates PI3K/Akt to upregulate the pro-survival protein Mcl-1, thereby overrides cells’ intrinsic apoptotic program [83]. This PI3K-dependent suppression of apoptosis permits prolonged survival of HCMV-infected monocytes, enabling viral dissemination throughout the host [84]. In addition, HCMV exploits PI3K/Akt signaling during both latency and reactivation. The viral GPCR US28, in concert with other latency-associated proteins, engages the Akt-mTOR axis to promote differentiation of CD34^+^ hematopoietic progenitors into monocytes, a prerequisite for viral reactivation [85]. Consistent with this mechanism, pharmacological inhibition of EGFR-PI3K signaling effectively blocks HCMV reactivation from latency, underscoring the virus’s reliance on host signaling pathways to orchestrate the switch between latent and lytic infection states [86].

Similarly, HHV-6A, another betaherpesvirus, robustly activates Akt-mTORC1 signaling in T cells, thereby reprogramming host cellular metabolism to favor viral replication. HHV-6A infection induces a Warburg-like metabolic shift leading to enhanced glucose uptake and lactate production through Akt/mTORC1 activation. The pharmacological blockade of this metabolic axis or mTORC1 activity markedly suppresses viral replication [87]. Although less well characterized, HHV-7, a closely related beta herpesvirus, is thought to employ analogous PI3K/Akt-dependent mechanisms to modulate cell survival and immune signaling. Notably, while PI3K/Akt activation generally facilitates herpesvirus immune evasion, for example, by suppressing pro-apoptotic and pro-inflammatory pathways, excessive pathway activation can paradoxically compromise effective antiviral immunity. This is illustrated by patients with activated PI3K-δ syndrome, in whom germline hyperactivation of PI3K signaling in immune cells is associated with recurrent and severe EBV and CMV infections [88]. Together, these observations underscore the necessity for herpesviruses to finely tune PI3K/Akt signaling sufficient to subvert host defenses yet restrained to avoid immune dysregulation.

### 5.2. PI3K/AKT Signaling in Herpesvirus-Mediated Oncogenesis

Chronic activation of PI3K/Akt signaling during persistent herpesvirus infection can contribute to virus-driven oncogenesis, most notably in the context of the oncogenic gammaherpesviruses EBV and KSHV/HHV-8. EBV encodes multiple latent proteins that constitutively engage the PI3K/Akt pathway, thereby promoting infected-cell survival, proliferation, and immune evasion [89]. A prototypical example is EBV latent membrane protein 1 (LMP1), a potent viral oncoprotein that functions as a constitutively active tumor necrosis factor receptor. In addition to robustly activating NF-κB and MAPK signaling, LMP1 directly engages PI3K through its cytoplasmic TRAF-binding domains, resulting in sustained Akt phosphorylation and activation [90]. This LMP1-driven Akt signaling upregulates pro-survival and metabolic programs and is essential for its oncogenic activity, as pharmacological inhibition of PI3K induces apoptosis in LMP1-expressing cells [91]. In parallel, EBV latent membrane protein 2A (LMP2A), a functional mimic of the B-cell receptor, delivers a constitutive survival signal in infected B cells by recruiting Lyn and Syk kinases as well as the p85 regulatory subunit of PI3K through its immunoreceptor tyrosine-based activation motif (ITAM) [92]. LMP2A-mediated activation of PI3K/Akt renders B cells refractory to normal antigen receptor signaling while simultaneously preventing apoptosis, thereby promoting the persistence and proliferation of EBV-infected cells. Collectively, these latency-associated mechanisms establish a pool of cells with constitutively active Akt and enhanced growth and survival capacity which is a critical step toward malignant transformation [93]. Consistent with this model, primary EBV-associated malignancies, including subsets of lymphomas and nasopharyngeal carcinoma, exhibit aberrant activation of PI3K/Akt signaling [94]. This sustained pathway activation has important downstream consequences; for example, LMP1-mediated Akt signaling inactivates the transcription factor FOXO3a, leading to repression of DNA repair genes such as *DDB1* [95]. The resulting defects in genomic maintenance promote genomic instability and mutation accumulation, further fueling oncogenesis. Thus, EBV exploits PI3K/Akt as a double-edged sword—supporting lifelong latency and cellular immortalization while simultaneously predisposing infected cells to malignant transformation.

KSHV/HHV-8 is the etiological agent of Kaposi’s sarcoma and several lymphoproliferative disorders. It also exploits the PI3K/Akt pathway leading to deep oncogenic consequences. A central viral effector is K1, a transmembrane protein containing a constitutively active immunoreceptor tyrosine-based activation motif (ITAM). K1 persistently engages the PI3K/Akt/mTOR axis; its expression in B cells induces Akt phosphorylation at both regulatory residues while concomitantly suppressing the PI3K antagonist PTEN [96]. Akt activation downstream of K1 sequesters pro-apoptotic FOXO transcription factors in the cytoplasm and downregulates Fas ligand expression at the cell surface, thereby potently inhibiting apoptosis and promoting cell survival. This K1-driven PI3K/Akt signaling is critical for KSHV fitness, enabling infected cells to withstand diverse cellular stress. For instance, during lytic reactivation, K1 protects infected cells from immune-mediated apoptosis, ensuring efficient viral replication. In endothelial cells, sustained K1-induced Akt/mTOR signaling promotes aberrant proliferation and angiogenic factor production, contributing directly to the tumorigenic phenotype characteristic of Kaposi’s sarcoma lesions. In addition to K1, KSHV encodes the viral G protein-coupled receptor vGPCR (ORF74), a constitutively active chemokine receptor that broadly activates host signaling cascades, including PI3K/Akt. vGPCR potently stimulates proliferative and survival pathways and, in experimental models, is sufficient to immortalize endothelial cells and induce highly vascular tumors that recapitulate key features of Kaposi’s sarcoma [97]. Consistent with these activities, KSHV-associated tumors exhibit sustained Akt activation, which is often further reinforced by viral microRNAs that suppress PTEN and other negative regulators of the pathway. The resulting chronic PI3K/Akt signaling not only prevents apoptosis but also remodels the tumor immune microenvironment-for instance, by inducing VEGF and IL-6 to drive angiogenesis and by upregulating PD-L1 to promote local immune evasion [98]. Collectively, constitutive Akt activation represents a defining hallmark of KSHV-driven malignancies, including primary effusion lymphoma and Kaposi’s sarcoma.

Across the herpesvirus family, the PI3K/Akt pathway emerges as a critical nexus of host–virus interaction acting truly as a double-edged sword. On one side, activation of PI3K/Akt is harnessed by herpesviruses to promote viral entry, replication, latency, and to suppress antiviral immune mechanisms (through enhanced cell survival and altered cytokine responses). On the other side, the chronic or dysregulated activation of this pathway can lead to pathological cell proliferation and malignant transformation in the host. The fact that several herpesvirus oncoproteins converge on PI3K/Akt underscores its importance in virus-mediated oncogenesis. These insights also highlight therapeutic opportunities: drugs targeting the PI3K/Akt/mTOR axis (some already in clinical use for cancers) have shown efficacy in inhibiting herpesvirus lytic replication and could potentially suppress virus-associated tumors. However, because PI3K/Akt signaling also plays key roles in normal immune function, therapeutics must strike a careful balance, much like the viruses themselves do, to exploit this pathway’s benefits while mitigating its dangers in host–virus interactions.

## 6. Therapeutic Implications

The PI3K/AKT signaling pathway contains multiple druggable nodes that have been extensively explored, predominantly in the context of cancer therapeutics. Table 1 summarizes active clinical trials targeting PI3K/AKT components, while Table 2 highlights key pathway targets and the corresponding pharmacological inhibitors under investigation.

### 6.1. Targeting the PI3K/AKT Pathway in Antiviral Strategies

Herpesviruses rely on the PI3K/Akt pathway explicitly and that makes it an appealing target for antiviral intervention. The disruption of this host signaling hub impairs multiple stages of viral life cycle from entry and gene expression to assembly, thereby leading to an effective suppression of infection. Therefore, a variety of studies have shown that pharmacologic inhibitors of PI3K/Akt can block lytic herpesvirus replication in cell cultures and animal models [99,100]. For example, broad spectrum PI3K inhibitors such as LY294002 or wortmannin were found to prevent HSV-1 from efficiently entering host cells and expressing key immediate-early genes [77]. Further, PI3K inhibition abrogates the actin remodeling needed for HSV’s membrane fusion and also reduces cell-to-cell spread. Notably, the PI3K inhibitor LY294002 also impairs the entry of beta- and gamma-herpesviruses, underscoring a conserved dependence on this pathway across the Herpesviridae family [77]. Similarly, varicella–zoster virus also requires an active PI3K/Akt/GSK-3β cascade for efficient replication and pharmacological inhibition of PI3K or Akt cause a severe drop in VZV replication and trigger apoptosis in infected cells [101]. Therefore, the host-targeted antivirals aiming at the PI3K/Akt axis can broadly curtail herpesvirus propagation by blocking critical host signals that the viruses hijack for their life cycles.

Importantly, many studies have identified specific points in the PI3K/Akt pathway that can be targeted to inhibit herpesviruses. For instance, HSV-1 infection is known to rapidly activate PI3K and its downstream kinase Akt; suppressing this activation (with PI3K inhibitors or dominant-negative mutants) leads to a sharp decline in HSV-1 immediate-early gene expression and progeny production. The treatment of HSV-1 infected corneal cells with an SGK1 kinase inhibitor or with the PI3K inhibitor LY294002 significantly decreased HSV-1 replication and virus-induced cell death. These findings underscore that host-targeted antivirals aimed at the PI3K/Akt axis can directly curtail herpesvirus propagation [102]. Alongside pharmacological inhibitors of PI3K/AKT axis that are precisely designed to target these proteins, numerous natural compounds also exhibit explicit potential to inhibit this pathway. For instance, natural compounds such as sophoridine exhibit potent anti-HSV-1 activity by downregulating PI3K/Akt signaling, leading to a significant reduction in the viral titre and protein production [99]. Similarly, alongside the active infection models, the blockade of downstream effectors of Akt has also proven effective in herpes simplex keratitis (HSK) models as well. Collectively, these examples show that disrupting the host PI3K/Akt axis, whether by synthetic inhibitors or by bioactive natural products can directly impede herpesvirus entry and replication in host cells.

Beyond its direct exploitation by herpesviruses, the PI3K/Akt pathway plays a central role at the cellular level in shaping macrophage biology during infection. In macrophages, PI3K/Akt signaling regulates survival, metabolic reprogramming, phagocytosis, and polarization states. Activation of PI3K/Akt downstream pattern-recognition receptors, Fc receptors, and cytokine receptors promote macrophage survival and limits premature apoptosis, allowing sustained innate immune surveillance at sites of herpes infection. Importantly, PI3K/Akt acts as a key switch between pro-inflammatory (M1-like) and immunoregulatory (M2-like) macrophage phenotypes by controlling NF-κB activity, glycolytic flux, and mTOR-dependent anabolic metabolism [103,104]. During herpes simplex virus infection, infiltrating macrophages represent one of the earliest and most abundant immune populations in infected tissues, including the cornea and nervous system. Dysregulated PI3K/Akt signaling in these cells can skew cytokine production, impair phagolysosomal maturation, and suppress type I interferon responses, thereby contributing to viral persistence, excessive inflammation, and tissue damage. Thus, PI3K/Akt signaling in macrophages serves as a critical cellular hub that balances antiviral defense and immunopathology during herpes pathogenesis, complementing its well-established proviral functions at the viral life-cycle level. We hypothesize that therapeutic strategies combining selective modulation of the PI3K/Akt pathway with macrophage-targeted interventions could provide synergistic control over herpesvirus infection and associated immunopathology [12]. While direct PI3K/Akt inhibition can restrict viral replication and survival signaling, its combination with macrophage-focused approaches may help rebalance innate immune responses at sites of infection. For example, partial or isoform-specific inhibition of PI3K/Akt could be paired with agents that promote antiviral macrophage polarization, enhance phagolysosomal maturation, or restore type I interferon signaling. Such combination approaches may limit excessive macrophage-driven inflammation while preserving their antiviral clearance capacity. Additionally, macrophage-targeted delivery systems, including nanoparticle-based drug carriers or ligand-directed inhibitors, could selectively modulate PI3K/Akt signaling within infiltrating macrophages, minimizing systemic toxicity. Together, these strategies could dampen viral persistence and tissue damage while maintaining effective innate immune surveillance, offering a rational framework for host-directed combination therapies during herpesvirus pathogenesis.

Beyond, in vitro experiments, there is encouraging preclinical and clinical evidence supporting PI3K/Akt pathway inhibition as an antiviral strategy. For instance, in InCMV infection, blockade of Akt’s downstream mTOR kinase (using rapamycin) has been shown to modestly reduce CMV replication [105]. More strikingly, in conditions where herpesviruses drive disease, PI3K/Akt inhibitors have demonstrated therapeutic benefit. Patients with EBV-driven lymphoproliferative disorders have responded to mTOR inhibitor therapy, with rapamycin aiding resolution of EBV-associated lesions along with the inhibition of development of EBV-positive B cell lymphomas in a mouse model [105]. In organ transplant recipients, switching immunosuppression from calcineurin inhibitors to sirolimus (an mTOR inhibitor) led to regression of Kaposi’s sarcoma lesions caused by KSHV [106]. These examples illustrate that repurposing PI3K/Akt pathway inhibitors, many of which are already approved for cancer or immunologic diseases, hold promise as a novel class of anti-herpesvirus agents [77]. Not only might this approach inhibit viral replication directly, but it could also target virus-induced malignancies, embodying a dual benefit in antiviral therapy.

### 6.2. Challenges and Opportunities in Therapy Development

The translation of PI3K/Akt-targeted strategies into safe and effective therapies comes with discrete challenges, as well as significant opportunities. The cellular function preserved for PI3K/Akt axis is vital for normal cell survival, metabolism, and immune function therefore its systemic inhibition often led to off-target toxicity. Thereby, the side effects associated with different PI3K/Akt inhibitors are very common with the potential of impacting immune system. In fact, clinical use of isoform-specific PI3K inhibitors (such as idelalisib for leukemia) has been associated with immunosuppression and opportunistic infection [107]. This highlights a double-edged sword: where inhibiting PI3K/Akt may hinder viruses, it may also impair antiviral immune responses or tissue homeostasis. Despite these hurdles, there are convincing opportunities for development of therapy. One is the wealth of existing drugs that modulate the PI3K/Akt/mTOR axis, which could be rapidly repurposed against herpesviruses [77]. These include PI3K inhibitors (some already in trials for immunological diseases like activated PI3K-δ syndrome), Akt inhibitors, and mTOR inhibitors that provide a toolkit to explore in antiviral applications. By leveraging isoform-specific inhibitors, it may be possible to achieve a degree of selectivity; for example, targeting the PI3K-δ isoform largely affects B and T lymphocytes, which could be advantageous in EBV or KSHV-driven lymphoproliferative diseases [108]. Another opportunity lies in combined modality approaches where PI3K/Akt inhibitors are not used as stand-alone antivirals but in concert with other treatments to enhance efficacy and mitigate risks. Notably, adjusting how these inhibitors are delivered can maximize benefits: localized or topical delivery (e.g., eye drops for HSK or a dermal gel for HSV lesions) could concentrate the antiviral effect at the site of infection while minimizing systemic exposure. Moreover, immunomodulatory benefits might be harnessed by careful dosing and partial inhibition of an overactive PI3K/Akt pathway might restore immune balance without completely crippling host defenses. For instance, patients with hyperactive PI3Kδ (APDS immunodeficiency) experienced improved T cell function when treated with a PI3Kδ inhibitor, without an increase in their chronic EBV or CMV infections [52]. This suggests a therapeutic window exists where host immunity can be bolstered by tempering an overactive PI3K/Akt pathway. In summary, while safety and specificity remain challenges, the ubiquity of the PI3K/Akt pathway in herpesvirus biology presents a unique opportunity to develop broad-spectrum host-directed antivirals, provided these therapies are engineered to strike a careful balance between antiviral potency and preservation of normal cell function.

### 6.3. Potential for Combination Therapies

The extent and complexities associated with herpesvirus infections involving active replication and latency establishment, combination therapies involving PI3K/Akt pathway modulators are a logical next step to enhance treatment outcomes. One strategy is combining a PI3K/Akt inhibitor with a traditional direct-acting antiviral drug. Targeting the virus on two fronts, directly by inhibiting viral enzymes (e.g., with acyclovir/foscarnet that target viral DNA polymerase) and simultaneously disrupting the pro-viral host signaling cascade, we could achieve synergistic suppression of replication. Such combinations might allow lower doses of each drug, reducing toxicity and delaying resistance. Although specific examples in the clinic are still forthcoming, the concept is supported by the observation that PI3K/Akt inhibitors act via mechanisms distinct from nucleoside analogs [99] and thus remain effective against strains resistant to standard antivirals. Another promising avenue is combining PI3K/Akt pathway inhibitors with other host-targeted therapies or immunotherapies. Because herpesviruses often disable immune responses, pairing a PI3K/Akt inhibitor with an immunomodulator might produce a one–two punch: the inhibitor undermines viral replication and survival signals, while the immunotherapy boosts the host’s ability to clear infected cells. In virus-associated cancers (such as EBV-positive lymphomas or KSHV-related tumors), this could translate to combining pathway inhibitors with immune checkpoint inhibitors or adoptive T-cell therapies to improve tumor (and virus) control. Early research in EBV-driven post-transplant lymphoproliferative disorder (PTLD) supports such an approach: dual blockade of the PI3K/Akt/mTOR axis has shown greater efficacy than either agent alone. In EBV+ lymphoma cells, PI3Kδ inhibition greatly augmented the anti-proliferative effect of mTOR inhibition (rapamycin), suggesting that hitting both the PI3K upstream and mTOR downstream nodes yields deeper suppression of virus-driven tumor cell growth [108]. This principle of multi-target combination could be extended to lytic infections as well, for example, using an Akt inhibitor together with an NF-κB inhibitor to simultaneously prevent the virus from hijacking two pivotal host pathways.

Finally, combination strategies can be designed to offset the limitations of PI3K/Akt inhibitors. Co-administering anti-inflammatory or tissue-protective agents might counteract any enhanced pathology (for example, mitigating ocular inflammation in HSK while the PI3K inhibitor limits viral replication). Conversely, prophylactic antivirals or immune stimulants could be used alongside PI3K/Akt blockade to prevent the potential reactivation of latent viruses. In essence, by carefully tailoring combination regimens, researchers aim to exploit the antiviral strengths of PI3K/Akt pathway inhibitors while compensating for their weaknesses. This might involve mixing systemic and topical therapies, alternating drug schedules, or pairing host-targeted and virus-targeted agents to achieve a more durable and safe suppression of herpesviruses. As research advances, such combination approaches offer a path to maximize the therapeutic benefit of targeting the PI3K/Akt pathway by truly capitalizing on its double-edged nature to tip the balance in favor of the host over the virus.

## 7. Conclusions

The PI3K/AKT signaling pathway has emerged as a central hub exploited by all HHVs to manipulate host cellular functions for their benefit. This evolutionarily conserved pathway supports viral entry, replication, latency, and reactivation through its regulation of cell survival, metabolism, and immune responses. Herpesviruses have developed sophisticated mechanisms to activate PI3K/Akt signaling at multiple stages of infection whether by mimicking growth factor signals, directly engaging pathway components, or altering host gene expression. While these interactions enable viral persistence and immune evasion, chronic or dysregulated activation of PI3K/AKT contributes to oncogenic transformation, particularly in the context of EBV and KSHV. Thus, the PI3K/AKT axis represents a double-edged sword: essential for viral success but also a driver of host pathology. Continued elucidation of these virus–host interactions offers valuable insight into herpesvirus pathogenesis and highlights potential targets for therapeutic intervention in both infectious and virus-associated malignant diseases.

## Figures and Tables

**Table 1:** Clinical trials actively evaluating therapeutic targeting of the PI3K/Akt pathway.

S.No.	Title	ClinicalTrials.gov ID	Drug
1.	An Umbrella Trial Based on Molecular Pathway for Patients with Metastatic TNBC. (FUTURE-SUPER)	NCT04395989	Drug: A1: Pyrotinib with nab-paclitaxel Drug: A2: nab-paclitaxel Drug: B1: everolimus with nab-paclitaxel Drug: B2: nab-paclitaxel Drug: C1: PD-1 with nab-paclitaxel and famitinib Drug: C2: nab-paclitaxel Drug: D1: VEGFR and nab-paclitaxel, with maintenance of VEGFR and capecitabine Drug: D2: nab-paclitaxel, with maintenance of capecitabine Drug: E1: everolimus with nab-paclitaxel Drug: E2: nab-paclitaxel
2.	Pediatric Patients Aged 4 to 11 Years With APDS	NCT05438407	Leniolisib
3.	Evaluating the Combination of Everolimus and Sorafenib in the Treatment of Thyroid Cancer	NCT01141309	Sorafenib with Everolimus
4.	Riluzole and Sorafenib Tosylate in Treating Patients with Advanced Solid Tumors or Melanoma	NCT01303341	Riluzole and Sorafenib
5.	Megestrol Acetate Compared with Megestrol Acetate and Metformin to Prevent Endometrial Cancer	NCT04576104	Drug: Extended-Release Metformin Hydrochloride Drug: Megestrol Acetate
6.	Safety Study of Adding Everolimus to Adjuvant Hormone Therapy in Women with High Risk of Relapse, ER+ and HER2- Primary Breast Cancer, Free of Disease After Receiving at Least One Year of Adjuvant Hormone Therapy	NCT01805271	Everolimus
7.	Onabotulinum Toxin A in Direct Brow Lift	NCT04383912	Onabotulinum Toxin A
8.	Testing the Addition of an Anti-cancer Drug, Copanlisib, to the Usual Immunotherapy (Nivolumab with or Without Ipilimumab) in Patients with Advanced Solid Cancers That Have Changes in the Following Genes: PIK3CA and PTEN	NCT04317105	Copanlisib, Nivolumab and Ipilimumab
9.	Z-Endoxifen Hydrochloride in Treating Patients with Metastatic or Locally Recurrent Estrogen Receptor-Positive Breast Cancer	NCT01327781	Endoxifen Hydrochloride
10.	Testing the Combination of Copanlisib, Nivolumab and Ipilimumab in Patients with Advanced Cancer and Lymphoma	NCT03502733	Copanlisib
11.	Testing the Addition of Copanlisib to Eribulin in Metastatic Triple Negative Breast Cancer	NCT04345913	Drug: Copanlisib Hydrochloride Drug: Eribulin Mesylate

**Table 2:** Target sites of selected PI3K pathway inhibitors.

S.No.	Class	Drug	Type
1.	Class IA PI3K	Alpelisib, Inavolisib, Risovalisib, Serabelisib, AZD8186, GSK2636771, Idelalisib, Linperlisib, Umbralisib, Copanlisib, Duvelisib, Taselisib	PI3K-isoform selective inhibitors
2.		STX-478, RLY-2608, LOXO-783, OKI-219	PI3Kα-mutant selective inhibitors
3.	Class IA PI3K Class IB PI3K	Buparlisib, Pilaralisib, Pictilisib	Pan-class I PI3K inhibitors
4.	mTORC1 and mTORC2	Apitolisib, Dactolisib, Gedatolisib, Omipalisib, Samotolisib, Voxtalisib	Dual pan-class I PI3K/mTOR inhibitors
5.	mTORC1	Everolimus, Ridaforolimus, Sirolimus, Temsirolimus	mTORC1- selective inhibitors
6.	AKT	Afuresertib, Capivasertib, Ipatasertib, MK-2206, Perifosine, Uprosertib, LY2780301, TAS0612	Pan-AKT inhibitors
7.	mTORC1/mTORC2	Vistuserib	

## Data Availability

No new data were created or analyzed in this study. Data sharing is not applicable to this article.

## Abbreviations

The following abbreviations are used in this manuscript:

HHV	Human Herpesvirus
PI3K	Phosphatidylinositol-3-kinase
AKT	Serine/threonine kinase
VZV	Varicella–zoster virus
EBV	Epstein–Barr virus
CMV	cytomegalovirus
PTEN	phosphatase and tensin homolog
KSHV	Kaposi’s sarcoma-associated herpesvirus
S6K	ribosomal S6 kinase
GSK3	Glycogen synthase kinase 3
TSC1-TSC2	tuberous sclerosis complex
mTORC1	mTOR complex 1
mTORC2	mTOR complex 2
